# Probing the Secrets of Alzheimer’s Disease Using Human-induced Pluripotent Stem Cell Technology

**DOI:** 10.1007/s13311-014-0326-6

**Published:** 2014-12-23

**Authors:** Lawrence S. B. Goldstein, Sol Reyna, Grace Woodruff

**Affiliations:** 1 Department of Cellular and Molecular Medicine, University of California, San Diego, 9500 Gilman Drive, La Jolla, CA 92093 USA; 2 Department of Neurosciences, University of California, San Diego, 9500 Gilman Drive, La Jolla, CA 92093 USA; 3 Biomedical Sciences Graduate Program, University of California, San Diego, 9500 Gilman Drive, La Jolla, CA 92093 USA

**Keywords:** Stem cells, Human neurons, Amyloid, hIPSC, Reprogramming

## Abstract

Our understanding of Alzheimer’s disease (AD) is still incomplete and, as a result, we lack effective therapies. Reprogramming to generate human-induced pluripotent stem cells provides a new approach to the generation of human neurons that carry the genomes of people with familial or sporadic AD. Differentiation of such stem cells to human neurons is already providing new insights into AD and molecular pathways that may provide new targets for effective therapy. These pathways include typical amyloid response pathways, as well as pathways leading from altered behavior of amyloid precursor protein to the elevated phosphorylation of tau protein. There is also a need for standardization of models so that isogenic lines differing only in the familial AD mutation can be compared.

**Electronic supplementary material:**

The online version of this article (doi:10.1007/s13311-014-0326-6) contains supplementary material, which is available to authorized users.

Alzheimer’s disease (AD) is a very common, currently incurable, and economically and emotionally costly disease. As such, it represents a major public health, scientific, and medical challenge. The literature around this disease is enormous, currently including > 100,000 papers, based on a recent Pubmed search. In spite of this enormous literature, and many sophisticated and careful experiments, understanding of AD remains an unsolved problem in 2 senses: first, because its molecular basis remains mysterious in many ways; and, second, by virtue of our current lack of a satisfyingly effective therapeutic.

The current major hypothesis in the field is the amyloid cascade model, which posits that oligomeric amyloid-beta (Aβ) fragments accumulate abnormally in patients with AD and drive neurotoxic events leading to neurodegeneration. While considerable evidence exists for the amyloid cascade model, experimental therapies built on this hypothesis have thus far been unsuccessful, and key details about how amyloid is neurotoxic either alone or in combination with other insults remain unclear [1].

One possible reason for our current situation may be that most mechanistic work thus far has relied either upon nonhuman animal models of AD or on experiments with human cells that are not actually neurons or glia. That these may be significant problems comes from 2 lines of thinking: first, non-neuronal cells lack many of the most important pathways that make neurons unique (including their ability to propagate an action potential, their large size, their extensive connectivity, their ability to convert an electrical signal into a chemical signal at neural synapses, and their extreme compartmentalization into biochemically distinct axonal and somatodendritic regions) and, second, euphemistically, humans are not just big mice [e.g., 2–4].

At present, no animal model appears to develop true AD and animal models often lack the range of phenotypes found in typical human AD. In fact, generating any of the Alzheimer’s-type pathology found in humans generally requires expression of one or more human genes in a mouse genetic background. That human genes are required at all for the development of typical pathology raises the question of whether the other, obviously mouse, genes may make a profound difference in the biochemical consequences and scientific interpretation of the lesions mimicked by expression of human familial AD (FAD) genes and mutations. Finally, of course, no animal model truly mimics the most common form of AD, which is sporadic AD (SAD).

One new opportunity that may provide insights into AD comes from recent advances in stem cell technology. In particular, reprogramming technology, which allows cells from patients with FAD or SAD to be reprogrammed to a pluripotent state to create human induced pluripotent stem cells (hIPSCs) is beginning to be of great value [5]. These hIPSC are valuable as they can be differentiated to a variety of cell types, such as neurons, astrocytes, oligodendrocytes, and other brain-derived cells. As these diverse cells carry the genomes of patients with AD, they can be used to test mechanisms of disease, to screen for potential drugs, and to evaluate functional roles of genetic variation observed in large genome-wide association studies. Furthermore, hIPSC technology allows investigators to identify and validate new pathways in the context of euploid human material in the absence of overexpression of key genes, which can bring its own artifacts.

Although this type of investigation of AD using hIPSC-derived models is still relatively new, a number of informative and provocative reports have appeared in the literature thus far, with more soon to come. The reports fall into 3 broad categories. First, are analyses of FAD mutations in *APP*; second are analyses of FAD mutations in *PS1* and *PS2*; and third are analyses and tests of Aβ toxicity on different types of human neurons.

## FAD *APP* Mutations

Several studies of hIPSC-derived human neurons carrying FAD *APP* mutations have appeared. The FAD *APP* mutations are present in 1 of the 2 genomic copies of *APP*, and are in the context of a “normal” euploid genome found in afflicted patients. Importantly, these mutations are in a normal *APP* gene in the absence of overexpression of amyloid precursor protein (APP) and with apparently normal genomic control of expression levels. Such studies have begun to provide new clues about early changes in neuronal physiology and function in response to mutations causing FAD.

One important study analyzed neurons made from hIPSC lines carrying FAD APP^E693delta^ and FAD APP^V717L^ mutations [6]. This work included an extensive analysis of Aβ production from these lines and found that APP^E693delta^ made less total Aβ while APP^V717L^ increased the Aβ42 : Aβ40 ratio with the increased ratio due to an increase in Aβ42 and no change in Aβ40. They noted that APP^E693delta^ cells accumulated Aβ oligomers inside the cell and exhibited signs of cellular stress that could be reversed by treatment with a drug previously reported to attenuate endoplasmic reticulum stress or reactive oxygen species generation, docosahexaenoic acid. Neurons carrying the genome of a patient with SAD in their analysis behaved the same as APP^E693delta^. These investigators did not report any analyses of tau protein phosphorylation or abundance.

A second study [7], from our laboratory, analyzed a hereditary APP duplication (APP^Dp^), which causes early-onset FAD. These investigators took advantage of the ability to purify neurons by fluorescence-activated cell sorting and observed that the APP^Dp^ neurons produced and secreted more Aβ40, exhibited elevated signs of elevated glycogen synthase kinase 3β activity, and exhibited elevated phosphorylation of tau protein at threonine 231, a proposed pathological site in tau protein. Strikingly, the elevated phosphorylation of tau protein was inhibited by the application of β-secretase but not γ-secretase inhibitors, suggesting that in this human neuronal system, aberrant phosphorylation of tau protein is driven, at least in part, by the β-C-terminal fragment fragment of APP. These investigators also observed an elevated frequency of enlarged endosomes similar to many previous reports of enlarged endosomes in non-neuronal cells caused by elevated expression of APP and by investigators studying postmortem brain material from Down syndrome fetuses, which carry an extra copy of *APP* by virtue of being trisomic for chromosome 21 [8–10]. In an analogous study [11], Down syndrome neurons were differentiated to a cortical fate and analyzed for production and deposition of Aβ, which were both observed. The researchers found elevated production of both Aβ40 and Aβ42 in neurons derived from both Down syndrome embryonic stem and induced pluripotent stem cells. In addition, elevated levels of total and phosphorylated tau protein with an apparent enrichment of tau protein were observed in the cell bodies of these neurons. Another recent report analyzed an APP^V717I^ mutation and found elevated Aβ42 and Aβ38 production, no change in Aβ40, elevated β-secretase cleavage, and elevated total and phosphorylated tau protein [12]. These investigators observed that Aβ antibody treatment in the culture medium suppressed the increased tau protein phenotype in these mutant neurons, suggesting that in this system Aβ production itself can drive elevated tau protein phenotypes.

Finally, Mertens et al. [13] generated hIPSC lines and neurons carrying an FAD APP^K724N^ mutation located in the cytosolic domain of APP. This mutation also exhibited elevated an Aβ42 : Aβ40 ratio caused by decreased Aβ40 and increased Aβ42. Surprisingly, these altered ratios could not be rescued by treatment with therapeutically relevant doses of nonsteroidal anti-inflammatory drugs (NSAIDS), which could alter these ratios in a variety of other cell types and transgenic models that overexpressed APP. The same NSAIDs had failed in clinical trials, even though results in transgenic models had been encouraging. These authors traced the likely failures to respond to therapeutically appropriate NSAID exposures to the normal levels of APP expression in the hIPSC-derived neurons and suggest that overexpression of APP in the models increased NSAID sensitivity as human neurons that overexpressed APP exhibited more similar NSAID responses to those in overexpression models. Thus, this hIPSC system more faithfully recapitulated human clinical responses to NSAIDs and suggests that future therapy development efforts should include assays of human euploid neurons as a key preclinical step.

Taken together, these studies suggest euploid human neurons with FAD *APP* mutations can reveal new insights regarding drug responses and perhaps other phenotypes. For example, different *APP* mutations share a common phenotype of alterations in tau protein modification, abundance, and localization. The possibility that these phenotypes are driven by a combination of exposure to Aβ and to other proteolytic intermediates of APP is also raised by these data.

## *PS1* and *PS2* Mutations 

A number of papers have reported analysis of presenilin (PS) mutations. Mutations analyzed include PS1^A246E^, PS1^A79V^, and PS2^N141I^ from patient material [13–15]. The PS1^dE9^ mutation was analyzed in our laboratory following induction by site-directed mutagenesis using transcription activator-like effector nuclease [16]. These studies all agree that the Aβ42: Aβ40 ratio is increased in all of these PS mutations. One study also reports significant gene expression changes in PS1^A246E^ mutations [14]. All of these reports are also remarkable by virtue of failing to observe any alteration in tau protein phosphorylation or abundance thus far in FAD PS mutations compared with APP mutations. While this difference could be a result of some unknown effect of culture conditions or timing, an interesting and testable possibility is that *APP* and *PS1* mutations differ in their initial phenotypes with respect to AD causation, which the hIPSC system has detected. Further work is needed to evaluate this important possibility. Woodruff et al. [16] also carefully examined the various allelic combinations of PS1^null^ and Ps1^dE9^ mutant-bearing neurons and concluded that null mutations and FAD mutations differed with respect to Aβ42 production and nicastrin maturation, and therefore that FAD *PS1* mutations were not equivalent to null mutations. PS1^L166P^ was analyzed in an overexpression system and was found to induce increases in the Aβ42 : Aβ40 ratio and altered responses to NSAID γ-secretase modulators (GSMs) [17]. Strikingly, human iPS-derived neurons overexpressing the PS1^L166P^ mutation or carrying the PS1^A79V^ mutation may be resistant to modulation by GSMs in the clinically relevant, low micromolar range. In fact, GSMs found to work in models overexpressing APP appeared to be ineffective in these human neurons [13]. Given that most NSAIDs cross the blood–brain barrier at levels < 30 μM and that clinical trials for NSAID γ-secretase modulators failed, it is telling that a human culture system was able to recapitulate the failure of mutant neurons to respond to these pharmacological interventions.

## Studies of Differentiation and Interactions with Aβ

A number of investigations have focused on inducing differentiation of human embryonic stem cells and human-induced pluripotent stem cells to neurons *in vitro* and characterizing their properties [18–23]. In addition, a number of the papers that have reported analysis of human mutations causing FAD have included comparisons of different neuronal differentiation systems. In summary, most of these studies generate neurons of different types, frequently cortical and enriched for glutamatergic in some cases. Some work has also focused on trying to generate basal forebrain cholinergic neurons [23]. Among the various papers, there is evidence that the neurons that are generated are compartmentalized appropriately with somatodendritic and axonal compartments, and that the neurons are capable of mounting action potentials. There is some diversity in how extensively electrophysiology was examined, how mature the neurons appeared to be, and how extensive the formation of synaptic contacts are. Clearly, considerable future work will focus on improving the *in vitro* differentiation systems and enhancing the maturity and network connectivity of neurons *in vitro*, perhaps including the addition of well-characterized astrocytes to promote general health and synapse formation.

Although APP processing during neurogenesis has been evaluated extensively in the mouse, in-depth analysis of the role of endogenous APP processing during differentiation and in different subtypes of iPSC-derived neurons is lacking. One important analysis of APP processing during neurogenesis reported protein and RNA levels of APP processing proteins measured at days 38, 45, and 52 [20]. It was found that full-length APP, APP cleaved by α-secretase APP cleaved by β-secretase, and Aβ species, as well as the β-secretase 1 enzyme increase in a time-dependent manner, but, strikingly, no changes were seen in the protein levels and expression of the γ-secretase complex [20].

Interestingly, *in vitro*-generated human neurons appear responsive to typical β- and γ-secretase inhibitors and modulators, but perhaps not at therapeutically relevant doses. Additional work will be needed to clarify this important issue. A few papers have also gone on to test whether Aβ is toxic to *in vitro*-generated human neurons, with initial indications being that this is true [18, 19]. In 1 case, selective toxicity of oligomeric Aβ42 to newly differentiating glutamatergic neurons was reported [18]. Effects of oligomeric or fibrillar species of Aβ_1-40_ and Aβ_1-42_ on nerve growth factor-stimulated embryonic stem cell neuronal differentiation were also reported [21]. In this study, the authors observed that both oligomeric forms of Aβ reduced the percentage of cholinergic neurons without affecting general neurogenesis or gliogenesis, but only Aβ_1-42_ selectively reduced the number of functionally mature neurons as assessed by evoked calcium imaging and Map2 staining (late neuronal marker). Interestingly, fibrillar forms of Aβ also suppressed cholinergic neuron differentiation, but had the added effect of enhancing gliogenesis (glial fibrillary acidic protein expression and staining). Though more research is necessary to determine how Aβ species affect neuronal differentiation, most discrepancies may be explained by differences in experimental methods, culture systems, and the nature of the Aβ species used.

## Some Closing Thoughts

Although the use of hIPSC technology is still in its infancy, early reports thus far have raised the possibility that this system may provide unique advantages for the discovery of factors that cause AD and how these factors respond to genetic or pharmacologic manipulation. One obvious possibility is that the generation of neurons carrying various FAD mutations or SAD genomes will provide a uniquely human neuronal system for the evaluation of potential therapeutic interventions. Early work has been valuable at understanding pathways in FAD neurons, but work on neurons with genomes from patients with SAD is just getting underway. Given the relatively high heritability of the risk of SAD, we anticipate that some novel phenotypes driven by the genomes of patients with SAD will be found, and genome-wide association study variants may be better understood, but more work is clearly needed before anything can be definitely said. Simultaneously, probing the human genetic and biochemical pathways in neurons, and eventually astrocytes and other cells [24], provide unique advantages owing to the likely differences between human and other organisms in neurons *versus* other cell types in their behavior. In this regard, of course, the failure thus far of therapies that work in the mouse or in non-neuronal human culture cells provides a cautionary signpost.

Moving forward, the opportunities for more complex culture systems in either 3 dimensions or with the addition of other defined cell types, such as astrocytes [24], oligodendrocytes [25], and microglia themselves carrying controllable genomes, is a provocative and exciting opportunity. One recent exciting entrant is a 3-dimensional model in which human neuronal stem cells that overexpress PS1 and APP mutants are differentiated in Matrigel [26]. Upon differentiation to neurons and other cell types, apparent amyloid plaque-like structures and neurofibrillary tangle-like structures were observed. This new system opens many new doors for testing mechanisms and pathways elucidated from euploid models and vice versa.

While the opportunities are exciting and unique, we have some suggestions for how to encourage experimental and intellectual rigor in these systems. Obviously, the likelihood that analysis of euploid cells carrying different mutations or genomes in the absence of high-level overexpression is a key advantage that some of these systems provide. We also suggest that working as much as possible in isogenic systems in which mutations can be compared in defined genetic backgrounds, as described in Woodruff et al. [16], could be very important given the known variability in human genomes and human physiology. As much as possible, we think that it would be advisable to work in cell lines that have been completely sequenced and a true diploid sequence determined to the level of the 1 genome described thus far [27], where enough work has been done to piece together the linkage and repulsion relationships of the resident haplotypes such that risk factor architecture is known and can be manipulated. We also think that, to the extent possible, investigators should work with cell culture systems in which the cell types present are well defined given the likely nonautonomous contributions of different cells to the behavior of the neurons. Thus, the combination of better differentiation systems that result in well-defined cell types and/or purification methods for different cell types during culture experiments may prove advantageous.

Finally, we note that a unique opportunity of the hIPSC system is the ability to probe how complex human genomic architectures predispose patients to AD and how they influence the behavior of various, participating cell types. In this regard, the reports that some genomes found in patients with SAD also generate *in vitro* culture phenotypes similar to those found in FAD mutations provides a striking example of how this may proceed. Thus, there is enormous promise in the utility of a human neuronal culture system to predict how individual genetic and cellular phenotypic variation contributes to response to pharmacological intervention at clinically relevant levels. Finally, the genetic technologies available with hIPSC may allow the deciphering of how complex genomic architectures found in individual humans act together to generate susceptibility and variation in response to the environmental factors that may also contribute to, or pharmacologically modify, AD phenotypes in patients.

## Electronic supplementary material

Below is the link to the electronic supplementary material.

ESM 1 (PDF 1.19 MB)
